# Bibliographical

**Published:** 1888-05

**Authors:** Eugene S. Talbot

**Affiliations:** Professor of Dental Surgery in the Womans’ College; Lecturer on Dental Pathology and Surgery in Rush Medical College, Chicago


### BIBLIOGRAPHICAL.

### Irregularities of the Teeth and their Treatment.

BY EUGENE S. TALBOT, M.D., D.D S.,

Professor of Dental Surgery in the Womans' College; Lecturer on Dental Pathology and Surgery in Rush Medical College, Chicago.

This work, recently issued from the press of P. Blakiston, Son & Co., of Philadelphia, is a most excellent compend upon the subject of Irregularities of the Teeth. In it is represented in a very clear and understandable manner the principles of the treatment of irregular teeth, with descriptions of the various appliances used for that purpose, and their application. The work, though less extensive than some others, is very practical indeed, not only for the practioner but is a very valuable work upon this subject for the student, and is better for this work than the larger works. Dr. Talbot was well prepared for the preparation of such a work by close study and investigation of the subject for many years. The principles which he lays down are well founded, and his principles of treatment are certainly efficient. The work should be in the hands not only of every practitioner but of every student as well. It can be obtained through any book seller.

